# Structure-Activity Relationship (SAR) Model for Predicting Teratogenic Risk of Antiseizure Medications in Pregnancy by Using Support Vector Machine

**DOI:** 10.3389/fphar.2022.747935

**Published:** 2022-02-25

**Authors:** Liyuan Kang, Yifei Duan, Cheng Chen, Shihai Li, Menglong Li, Lei Chen, Zhining Wen

**Affiliations:** ^1^ College of Chemistry, Sichuan University, Chengdu, China; ^2^ Department of Neurology, West China Hospital, Sichuan University, Chengdu, China; ^3^ Medical Big Data Center, Sichuan University, Chengdu, China

**Keywords:** drug safety evaluation, structure-activity relationship, machine learning, antiseizure medications, drug-induced teratogenesis

## Abstract

Teratogenicity is one of the main concerns in clinical medications of pregnant women. Prescription of antiseizure medications (ASMs) in women with epilepsy during pregnancy may cause teratogenic effects on the fetus. Although large scale epilepsy pregnancy registries played an important role in evaluating the teratogenic risk of ASMs, for most ASMs, especially the newly approved ones, the potential teratogenic risk cannot be effectively assessed due to the lack of evidence. In this study, the analyses are performed on any medication, with a focus on ASMs. We curated a list containing the drugs with potential teratogenicity based on the US Food and Drug Administration (FDA)-approved drug labeling, and established a support vector machine (SVM) model for detecting drugs with high teratogenic risk. The model was validated by using the post-marketing surveillance data from US FDA Spontaneous Adverse Events Reporting System (FAERS) and applied to the prediction of potential teratogenic risk of ASMs. Our results showed that our proposed model outperformed the state-of-art approaches, including logistic regression (LR), random forest (RF) and extreme gradient boosting (XGBoost), when detecting the high teratogenic risk of drugs (MCC and recall rate were 0.312 and 0.851, respectively). Among 196 drugs with teratogenic potential reported by FAERS, 136 (69.4%) drugs were correctly predicted. For the eight commonly used ASMs, 4 of them were predicted as high teratogenic risk drugs, including topiramate, phenobarbital, valproate and phenytoin (predicted probabilities of teratogenic risk were 0.69, 0.60 0.59, and 0.56, respectively), which were consistent with the statement in FDA-approved drug labeling and the high reported prevalence of teratogenicity in epilepsy pregnancy registries. In addition, the structural alerts in ASMs that related to the genotoxic carcinogenicity and mutagenicity, idiosyncratic adverse reaction, potential electrophilic agents and endocrine disruption were identified and discussed. Our findings can be a good complementary for the teratogenic risk assessment in drug development and facilitate the determination of pharmacological therapies during pregnancy.

## Introduction

Congenital malformations are defined as the malformations of organs or body parts during development *in utero*, mainly attributed to hereditary, maternal, external environmental or some unknown factors. Major congenital malformations (MCMs) are defined as structural abnormalities with surgical, medical, functional, and or cosmetic importance (Tomson et al., 2019). It is not only an irreparable blow to the family, but also a burden on society. As a potential teratogen, a number of drugs can pass through the placental barrier and affect the growth and development of the fetus *in utero*. It is commonly accepted that the most sensitive period to teratogens is during active organogenesis, which is from three to 8 weeks after fertilization. Some organs, such as the brain, will continue to be very active developmentally after active organogenesis and may still be affected by teratogens (Ornoy, 2009).

Antiseizure medications (ASMs) are used to control various types of convulsive disorders or as mood stabilizers. About 0.3–0.7% of pregnant women are diagnosed with epilepsy (Viinikainen et al., 2006), most of which receive ASMs monotherapy (74%) (Meador et al., 2018). Although the incidence of MCMs in offspring of women with epilepsy (WWE) receiving ASMs monotherapy during pregnancy is very low (4%), the rate is still higher than that of MCMs in the general population (Campbell et al., 2014). In clinical practice, the lowest effective dose is usually recommended for seizure control for the safety of both mother and child. Furthermore, WWE should avoid unexpected pregnancies because ASMs will take a period of time to metabolize to a safe level for embryo. Therefore, the teratogenic risk evaluation of ASMs is crucial for the clinical medications.

During the last decade, data from large prospective epilepsy and pregnancy registries have been used to compare the incidence and the risk of MCMs in offspring exposed to different ASMs. When exposed to different antiseizure medications (ASMs), the incidence of teratogenesis exhibits significant difference. The incidence of MCMs in offspring exposed to valproate and phenobarbital is 5–11% and 6–7%, respectively, while it is only 2–3% for lamotrigine and levetiracetam (Brodie, 2006; Hernandez-Diaz et al., 2012; Tomson and Battino, 2012; Bromley et al., 2017; Tomson et al., 2018). Fetal exposure to ASMs is associated with an increased risk of MCMs. Although there is a variation in the rate, the evidence in pregnancy registries implies that valproate is associated with the highest risk of MCMs among ASMs. The post-marketing surveillance data in US Food and Drug Administration (FDA) Spontaneous Adverse Events Reporting System (FAERS) also indicates the high prevalence of congenital malformations induced by valproate. (Supplementary Table S1). In addition, carbamazepine, phenobarbital, phenytoin, and topiramate are also associated with the increased risk of MCMs in offspring, while lamotrigine and levetiracetam are of relatively low teratogenic risk (Tomson et al., 2011; Hernandez-Diaz et al., 2012; Campbell et al., 2014; Tomson et al., 2018). In preclinical studies, zebrafish embryo model and the animal models have also been established to evaluate the developmental toxicity, and teratogenic activity of the drugs (Murayama et al., 2018; Chetot et al., 2020; Jarque et al., 2020; Nguyen et al., 2021; Simeon et al., 2021). In addition, a few *in silico* models have been proposed to predict the reproductive toxicity of the chemical compounds (Basant et al., 2016; Jiang et al., 2019), but none of them is developed for the purpose of teratogenic risk prediction.

**FIGURE 1:**
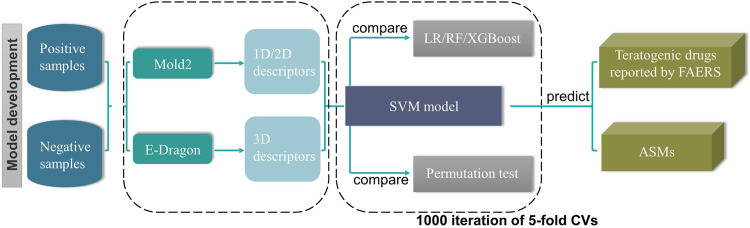
The workflow of our study.

Although large scale epilepsy pregnancy registries played an important role in evaluating the teratogenic risk of ASMs, the teratogenic risks of a large proportion of ASMs, especially the newly approved ones, are still unknown because of the limited post-marketing surveillance data. To fill this gap, we endeavoured to construct a model to predict the potential teratogenic risk of the drugs based on their chemical structural information. The detailed workflow was depicted in Figure 1.

## Materials and Methods

### Generation of Curated Dataset

FDA-approved drug labeling contains the essential information for the safe and effective use of the drug, which is a relative accurate and stable data source to evaluate the teratogenic risk of a drug (Wu et al., 2019). Drug labeling with keywords “teratogenicity” were extracted from FDALabel public version (https://nctr-crs.fda.gov/fdalabel/ui/search) (Data was acquired on April 26th, 2021) by using full text searching. In total, we obtained 7,926 drug labeling with keywords “teratogenicity”. Then, we kept 7,059 drug labeling with single active ingredient based on Unique Ingredient Identifier (UNII). Subsequently, we only kept the drug labeling administered through oral or parenteral route (6,180 drug labeling). If multiple drug labeling contains the same UNII, we kept the latest version of drug labeling. Consequently, we generated a curated dataset containing 286 drugs with potential teratogenic risk.

Based on the FDA use-in-pregnancy ratings, we divided the 286 drugs into high teratogenic risk (positives, 67 drugs) group and low teratogenic risk (negatives, 45 drugs) group based on the drug labeling information. A drug was categorized into high teratogenic risk if it was indicated in drug labeling that adequate and well-controlled studies or animal studies had shown the teratogenic risk to the foetus, while a drug was categorized into low teratogenic risk if both animal and human studies showed no risk to the foetus. In addition, we further validated teratogenic risk of the drugs in low teratogenic risk group by using the post-marketing surveillance data in FAERS.

To generate the structure data files (SDFs) of the drugs, we mapped the drugs to the PubChem (https://pubchem.ncbi.nlm.nih.gov/) (Kim et al., 2016) database and obtained the 2D and 3D SDFs of 112 drugs for SAR model construction, among which 67 drugs were positives and 45 drugs were negatives.

### FDA FAERS Dataset

The FDA FAERS database (https://open.fda.gov/data/faers/) contains the post-marketing surveillance data of human prescription drugs and is commonly used for drug safety surveillance. Current version of FAERS database contains several hurdles including nonstandard usage of terms, duplicate records, and mapping of drugs and adverse events inaccurately. Following the preprocessing procedures proposed by Banda et al. (2016), we compiled the records in FAERS from 1 Jan 2004 to 31 Dec 2020 via the four steps: 1) removing the duplicated cases based on four demographic data fields, namely event date, age, sex, and reporter country; 2) mapping the drug names into RxNorm CUIs and Observational Health Data Sciences and Informatics (OHDSI) standard vocabulary concept identifiers; 3) mapping the drug indications and reactions from MedDRA to the Systematized Nomenclature of Medicine Clinical Terms (SNOMED-CT) standard codes by using OHDSI vocabulary; 4) establishing the drug-event pair by linking each drug in each case with the associated outcome and calculating the reporting odds ratio (ROR) for each drug by the equation:

$$\mathrm{ROR}=\frac{\mathrm{a/c}}{\mathrm{b/d}}$$ (1)

where *a*, *b*, *c* and *d* were the number of cases defined by the contingency table (Table 1).

**TABLE 1:** Contingency table for the calculation of reported odds ratio.

	Cases with current ADR	Cases without current ADR
Cases with current drugs	a	b
Cases with other drugs	c	d

We retrieved the cases and obtained the drugs with teratogenic risk according to the following steps. Firstly, all the preferred terms related to congenital malformation under the subject word “Congenital Abnormalities” in MeSH were extracted (Supplementary Table S2). Secondly, we used these preferred terms as keywords to search the FAERS database and kept the cases if the preferred terms were reported as adverse events. Finally, the drug reported as the primary suspected drug in each case were considered as the teratogenic risk drug. After removing the drugs that existed in the training set and keeping the drugs only administered through oral or parenteral route, we kept drugs, for which the number of reported cases was greater than three and the lower limit of the 95% CI was greater than 1. We uploaded them into the PubChem database to generate 2D and 3D SDFs. In addition, the Anatomical Therapeutic Chemical (ATC) code for each drug was extracted from the Drugbank database (https://go.drugbank.com/) (Wishart et al., 2008).

### Calculation of Molecular Descriptors

The 1D and 2D molecular descriptors were calculated by Mold2 (https://www.fda.gov/science-research/bioinformatics-tools/mold2) with the 2D SDFs of the drugs, which is a free and easy-to-use software package (Hong et al., 2008). The 3D molecular descriptors were calculated by E-Dragon web server (http://www.vcclab.org/lab/edragon/) (Mauri et al., 2006) with 3D SDFs of the drugs.

### Model Development

Support vector machine (SVM) algorithm, which is a classical machine learning method that maps vectors nonlinearly to a high-dimensional space and constructs decision surfaces in the high-dimensional space (Cortes and Vapnik, 1995), was proposed in this study for the teratogenic risk prediction. We used the radial basic function (RBF) as the kernel function and optimized two parameters, namely, regularization parameter (c) and kernel width (γ), by grid searching and five-fold cross-validation. The optimal regularization parameter (c) and kernel width (γ) were 2 and 0.001, respectively. After determining the optimal parameters, we performed 1,000 iterations of 5-fold cross-validation to investigate the performance of the models. For comparison, we constructed the models by using logistic regression (LR), random forest (RF), and extreme gradient boosting (XGBoost). LR is a classical linear classifier with a sigmoid activation function (Park, 2013). The inverse of regularization strength (C) was optimized in modeling procedure. RF is an ensemble classifier, which integrates multiple decision trees (DT) and then classifies the samples according to the voting results (Qi, 2012). Three parameters, namely the number of trees, the number of features and the maximum depth of a tree were optimized in our study. XGBoost is also an ensemble classifier and is integrated with multiple gradient boosted decision trees (Chen and Guestrin, 2016). Different from RF, XGBoost establishes the association between the decision trees and can be considered as a scalable end-to-end tree boosting system. Two parameters, namely minimum loss reduction parameter (γ) and the maximum depth were optimized in our study. The modeling procedures and predictions were conducted in Python 3.7.5 with the package *scikit-learn 0.24.1*. All the data and source codes can be freely downloaded from Github (https://github.com/LiSH7450/DIT_model).

### Performance Evaluation

To evaluate the performance of the models, nine performance metrics, namely accuracy, recall rate, precision, specificity, Matthews correlation coefficient (MCC), balance accuracy (BACC), F1 score, the area under the receiver operating characteristic curve (AUROC), and the area under the precision-recall curve (AUPRC), were used in this study. The equations of the nine metrics were listed as follow:

$$\mathrm{accuracy=}\frac{\mathrm{TP+TN}}{\mathrm{TP+TN+FP+FN}}$$ (2)

$$\mathrm{recall}\;\mathrm{rate=}\frac{\mathrm{TP}}{\mathrm{TP+FN}}$$ (3)

$$\mathrm{precision=}\frac{\mathrm{TP}}{\mathrm{TP+FP}}$$ (4)

$$\mathrm{MCC=}\frac{\mathrm{TP\times TN-FP\times FN}}{\sqrt{\left(\mathrm{TP+FP}\right)\left(\mathrm{TP+FN}\right)\left(\mathrm{TN+FP}\right)\left(\mathrm{TN+FN}\right)}}$$ (5)

$$\mathrm{BACC=}\frac{\left(\mathrm{TPR+TNR}\right)}{2}$$ (6)

$$F1\mathrm{score=}\frac{\mathrm{2\times}\left(\mathrm{precision\times recall}\;\mathrm{rate}\right)}{\mathrm{precision+recall}\;\mathrm{rate}}$$ (7)

$$\mathrm{AUROC=}\overset{1}{\underset{\mathrm{x=0}}{\int }}\mathrm{TPR}\left(\mathrm{FP}R^{\mathrm{-1}}\left(x\right)\right)\mathrm{dx}$$ (8)

$$\mathrm{AUPRC=}\int_{-\infty }^{\infty }\mathrm{precision}\left(x\right)\mathrm{dP}\left[Y\leq x\right]$$ (9)

$$\mathrm{specificity=}\frac{\mathrm{TN}}{\mathrm{TN+FP}}$$ (10)

## Results

To facilitate the prevention and early detection of drug-induced teratogenesis in pregnancy, we generated a curated dataset and developed a SAR model to predict the potential teratogenic risk of a drug. The model was subsequently applied to evaluating the teratogenic risk of ASMs.

### The SAR Model Performance

In total, we generated 777 1D and 2D molecular descriptors and 1,666 3D molecular descriptors. After filtering the descriptors with the standard deviation across all samples less than 0.001, we finally kept 2083 1D/2D/3D descriptors as features for model construction. Of the 2083 molecular descriptors, 585 were 1D/2D molecular descriptors and 1,498 were 3D molecular descriptors.

**FIGURE 2:**
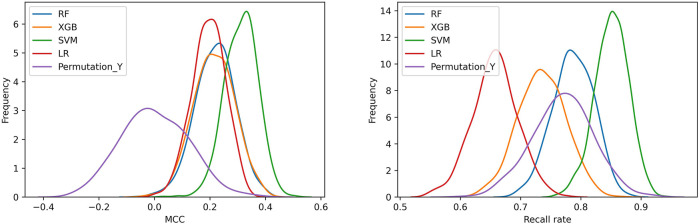
The distribution of MCCs and recall rates achieved by SVM, LR, RF, and XGBoost with 1,000 iterations of five-fold cross-validation. **(A)** The distribution of MCCs. **(B)** The distribution of recall rates. Permutation_Y indicated that the SVM models constructed by using the samples with randomly permutated labels.

The frequency distribution of MCCs and recall rates achieved by 1,000 iterations of five-fold cross-validation of the four models and the random model was shown in Figure 2. Either for MCC or recall rate, the performance of four models was obviously higher than that of random model. The MCCs and recall rates achieved by SVM were higher than those achieved by LR, RF and XGBoost. Table 2 showed the average values of performance metrics and the corresponding standard deviations of 1,000 iterations. Among the four modeling algorithms, SVM exhibited the best performance on predicting the teratogenic risk. The average MCC, BACC, F1 score, AUROC, AUPRC, specificity and recall rate achieved by SVM were 0.312, 0.640, 0.762, 0.640, 0.676, 0.428, and 0.851, respectively. Specifically, the average recall rate was 0.851 achieved by SVM significantly higher than those achieved by LR, RF, and XGBoost (average recall rates = 0.657, 0.787 and 0.735 for LR, RF, and XGBoost, respectively. Welch’s *t*-test *p*-values < 0.0001, respectively), indicating that SVM performed better on detecting the drugs with high teratogenic risk.

**TABLE 2:** The prediction results of SVM, LR, RF, XGBoost and Permutation_Y*.

	SVM	LR	RF	XGBoost	Permutation_Y
Accuracy	0.681 ± 0.001	0.610 ± 0.001	0.638 ± 0.001	0.630 ± 0.001	0.554 ± 0.002
Recall rate	0.851 ± 0.001	0.657 ± 0.001	0.787 ± 0.001	0.735 ± 0.002	0.770 ± 0.003
Precision	0.690 ± 0.000	0.680 ± 0.001	0.668 ± 0.001	0.676 ± 0.001	0.599 ± 0.001
MCC	0.312 ± 0.004	0.195 ± 0.004	0.219 ± 0.005	0.215 ± 0.005	0.001 ± 0.015
BACC	0.640 ± 0.001	0.598 ± 0.001	0.602 ± 0.001	0.604 ± 0.001	0.501 ± 0.003
F1 score	0.762 ± 0.000	0.668 ± 0.001	0.722 ± 0.001	0.704 ± 0.001	0.673 ± 0.001
AUROC	0.640 ± 0.001	0.598 ± 0.001	0.602 ± 0.001	0.604 ± 0.001	0.501 ± 0.003
AUPRC	0.676 ± 0.000	0.653 ± 0.000	0.653 ± 0.000	0.656 ± 0.000	0.600 ± 0.001
Specificity	0.428 ± 0.003	0.539 ± 0.002	0.416 ± 0.004	0.474 ± 0.004	0.233 ± 0.006

*Permutation_Y, the SVM model constructed by using the samples with permutated lables.

### Teratogenic Risk Prediction of FAERS Dataset

**FIGURE 3:**
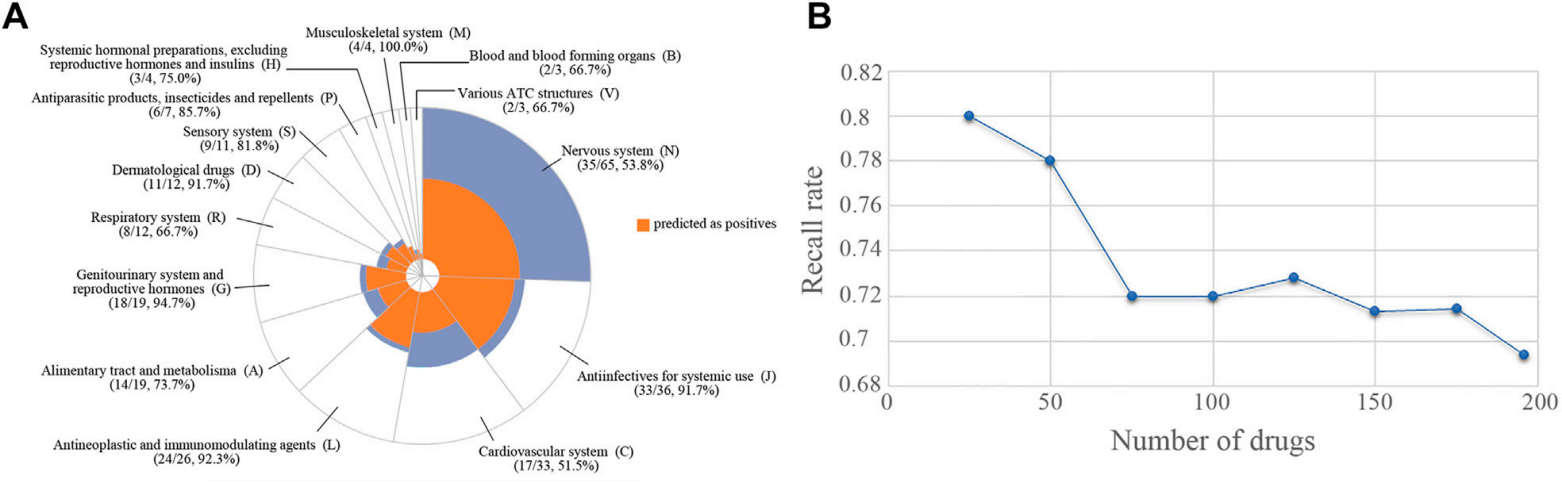
The distribution of the drugs with teratogenic risk in different therapeutic categories in FAERS and the recall rates obtained by SVM model. **(A)** Distribution of all the drugs and the drugs predicted as positives. **(B)** The trend of recall rate changed with the number of drugs increased.

The SVM model was validated by using 196 drugs that were reported to induce congenital malformations in the FDA FAERS database. The distribution of the drugs in different therapeutic categories was shown in Figure 3A. The Anatomical Therapeutic Chemical (ATC) code for each drug was extracted from the Drugbank database by searching for the drug names. There is no therapeutic category associated with two of 196 drugs. 37 drugs are associated with more than one therapeutic category. For each of the 37 drugs, we counted it once in each of the categories it belongs to when calculating the recall rate of our model for each of the therapeutic categories. Among 196 drugs, over half percent of the drugs belonged to four therapeutic categories including nervous system (N, 65 drugs, 53.8%), antiinfectives for systemic use (J, 36 drugs, 91.7%), cardiovascular system (C, 33 drugs, 51.5%), and antineoplastic and immunomodulating agents (L, 26 drugs, 92.3%). A total of 136 drugs (136/196, 69.4%) were predicted to be high teratogenic risk by the SVM model. For each therapeutic category, the SVM model can detect more than 50% drugs (Figure 3A). Specifically, for the therapeutic category of antineoplastic and immunomodulating agents (L), 92.3% drugs were correctly predicted with potential teratogenic risk.

Subsequently, the 196 drugs were ranked by their RORs in descending order and were used to investigate the performance of the SVM model on detecting high teratogenic risk drugs. We generated a subset containing the top 25 drugs and then extended it by adding 25 drugs at a time until all drugs were included. For each subset, we calculated the recall rate achieved by SVM. Figure 3B showed the recall rates for all the subsets. It can be seen that the SVM model can detect 80.0% drugs from the top 25 drugs. The recall rate decreased with the increase of the number of drugs involved in the subset. Only 69.4% drugs can be detected in the top 196 drugs, indicating that the SVM model was more sensitive to detecting the drugs with high teratogenic risk. In addition, we investigated the prediction results of the ten drugs ranked at the top of the subset and found that 8 of them (80%) were correctly predicted (Table 3).

**TABLE 3:** The information of top ten drugs with high teratogenic risk in FAERS.

Drug name	Odds ratio	ATC code
cisapride*	364.7	Withdrawn
Levamisole	284.7	Withdrawn
bedaquiline*	146.7	J04AK05
ibutilide*	139.3	C01BD05
ondansetron*	95.0	A04AA01
clofazimine*	78.5	J04BA01
Nelfinavir	77.0	J05AE04
folic acid*	70.7	V04CX02; B03BB01
Procainamide	64.1	C01BA02
Vandetanib*	63.8	L01XE12

*Drugs that were predicted as positives by our model.

### Teratogenic Risk Prediction of ASMs

**FIGURE 4:**
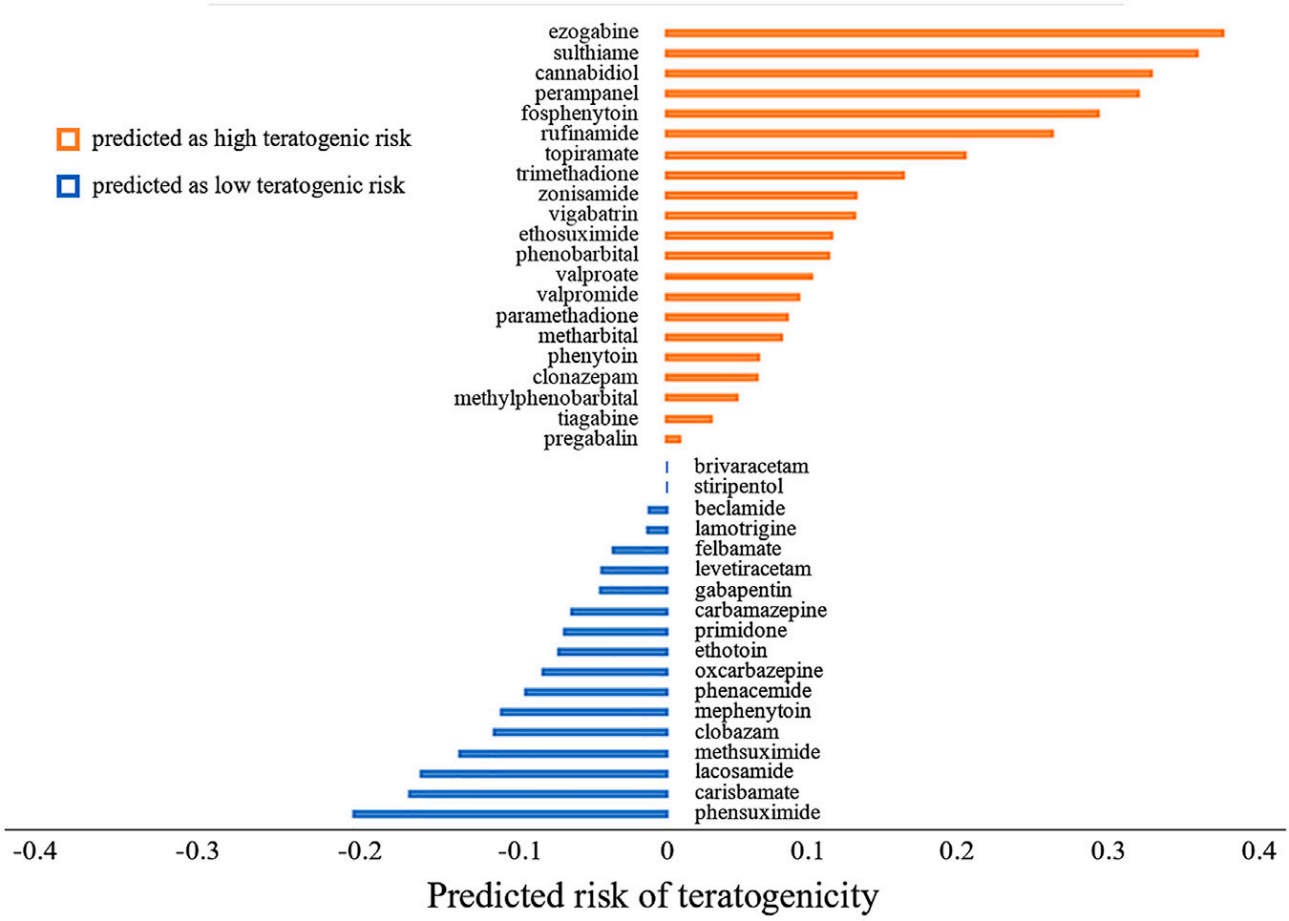
The predicted risk of teratogenicity of 39 antiseizure medications. The antiseizure medications were ranked by their predicted probabilities in descending order. The higher the predicted probability value is, the higher the teratogenic risk is. We took 0.5 as a cutoff. A drug with predicted probability larger than 0.5 was predicted as high teratogenic risk, otherwise it was predicted as low teratogenic risk. For the sake of demonstration, we subtracted 0.5 from the predicted probability of each drug before plotting.

The predicted probabilities of ASMs were shown in Figure 4. We took 0.5 as a cut-off of predicted probability to classify the drugs. The drugs with predicted probability larger than 0.5 were considered as high teratogenic risk drugs. It can be seen from the figure that 21 ASMs (21/39, 53.8%) including four commonly used ASMs, namely valproate, topiramate, phenobarbital, and phenytoin, were predicted to be high teratogenic risk. In addition, five commonly used ASMs including lamotrigine, levetiracetam, gabapentin, carbamazepine, and oxcarbazepine were predicted to be low teratogenic risk.

## Discussion

Although the incidence of drug-induced malformations accounts for only 1% or less of the cases of birth defects with known causes (De Santis et al., 2001), the teratogenicity of drugs has always been a widespread concern in clinical medication. Since the first case report indicated that ASMs were associated with congenital malformations in the 1960s (Janz, 1964; Meadow, 1968), a number of efforts had been made for assessing the teratogenic risk of ASMs, so as to help pregnant women avoid the harm of drugs to the offspring while treating epilepsy (Tomson et al., 2011; Hernandez-Diaz et al., 2012; Campbell et al., 2014; Thomas et al., 2017; Tomson et al., 2018; Tomson et al., 2019). Case-controlled studies and animal studies are currently reliable approaches to evaluate the teratogenic risk of ASMs. The teratogenicity of commonly used ASMs including valproate, carbamazepine, and lamotrigine had been systematically investigated by reviewing the prevalence of major congenital malformations in epilepsy pregnancy registries (Tomson et al., 2011; Hernandez-Diaz et al., 2012; Campbell et al., 2014; Tomson et al., 2018; Tomson et al., 2019). However, for a large proportion of ASMs, especially the newly approved ones, the lack of evidence is a big hurdle to effectively estimate the teratogenic risk. To fill this gap, we proposed a SAR model to detect the drugs with high teratogenic risk and applied it to assess the teratogenic risk of ASMs. The model was constructed by using SVM algorithm with the chemical structural descriptors of the drugs as features.

As a result, the SVM model performed the best when compared to the state-of-art machine learning approaches (Figure 2). The average MCC, F1 score and recall rate for classifying the teratogenic risk of drugs in the curated dataset were 0.312, 0.762, and 0.851, respectively. When validated by the FAERS dataset, the SVM model detected 136 of 196 drugs with teratogenic risk (recall rate = 0.694). The distribution of the predicted probability of the teratogenic risk of the drugs were shown in Supplementary Figure S1. For the subsets of top ten and top 25 drugs ranked by their RORs, the recall rates achieved by the SVM model were 0.700 and 0.800, respectively, indicating that the model was more sensitive to detecting the high teratogenic risk drugs (Figure 3B). Interestingly, the model seems to be sensitive to detecting the teratogenic risk drugs in two therapeutic categories, namely antiinfectives for systemic use and antineoplastic and immunomodulating agents, for which the recall rates were 91.7% and 92.3% (Figure 3A).

For the risk prediction of ASMs, valproate, topiramate, phenobarbital and phenytoin were predicted to be high teratogenic risk, which were consistent with drug labeling information, and reported prevalence in pregnancy registries (Tomson et al., 2011; Hernandez-Diaz et al., 2012; Campbell et al., 2014; Tomson et al., 2018; Tomson et al., 2019). The FDA-approved drug labeling of these four drugs included clear statement of the teratogenic risk of use during pregnancy. Valproate was warned in the BOXED WARNING labeling section that it can cause major congenital malformations, e.g., neural tube defects, and should avoid treating women with epilepsy during pregnancy. The teratogenic severity of topiramate, phenobarbital and phenytoin was also indicated in WARNINGS AND PRECAUSIONS labeling section that infants exposed to these drugs *in utero* had an increased risk for congenital malformations and other developmental outcomes. Especially for topiramate and phenobarbital, case-controlled studies and the data in pregnancy registries indicated that the use of these two drugs might increase the risk of fetal abnormalities. Furthermore, relatively high prevalence of major congenital malformations of valproate, topiramate, phenobarbital, and phenytoin is reported in the pregnancy registries (Tomson et al., 2011; Hernandez-Diaz et al., 2012; Campbell et al., 2014; Thomas et al., 2017; Tomson et al., 2018; Tomson et al., 2019). In addition, five commonly used ASMs including lamotrigine, levetiracetam, gabapentin, carbamazepine, and oxcarbazepine were predicted to be low teratogenic risk, which was also consistent with the low reported prevalence of major congenital malformations in pregnancy registries (Tomson et al., 2011; Hernandez-Diaz et al., 2012; Thomas et al., 2017; Tomson et al., 2018; Tomson et al., 2019).

The chemical structures of drugs containing the toxicophores (or structural alerts (SAs)) was considered as one of the important factors of drug-induced teratogenesis (Sankar and Lerner, 2008). For instance, the aromatic ring in the chemical structure of a drug, e.g., lamotrigine, may produce a highly reactive epoxide or arene oxide in the metabolism through cytochrome P-450 family of enzymes and generate reactive metabolites, which may contribute to teratogenesis (Sankar and Lerner, 2008). To further investigate the structural features of ASMs and help better understand the mechanisms of teratogenicity, the SAs associated with known toxic property were extracted from the structures of 39 ASMs. We uploaded the SDFs of the ASMs into the Online Chemical Modeling Environment (OCHEM, https://ochem.eu/) (Sushko et al., 2011) and used a web server named ToxAlerts (Sushko et al., 2012) for mapping the toxicological SAs. Consequently, a total of 23 SAs that might be associated with teratogenicity were extracted, which belonged to four endpoints, namely genotoxic carcinogenicity and mutagenicity, idiosyncratic adverse reaction, potential electrophilic agents and endocrine disruption (Table 4). The chemical structure of the common antiepileptic drug valproate contained the carboxylic acids, which might lead to the formation of reactive metabolites, and then induce the dose-dependent idiosyncratic adverse reaction (Kalgutkar and Soglia, 2005). Another drug phenytoin contained a toxicological alert ethane-1,1-diyldibenzene, which was reported to be of potential estrogenic and androgenic activities (Nendza et al., 2016). In addition, ezogabine was predicted to be a high teratogenic risk drug by the SVM model with the highest predicted probability (0.858), for which the chemical structure contained three SAs. One of the SA was anilines, which might form the reactive metabolites by bioactivation and was linked to the idiosyncratic adverse reaction (Kalgutkar and Soglia, 2005). The other two SAs were aromatic amines and derivatives of urethane, which were reported to be associated with genotoxic carcinogenicity and mutagenicity (Ashby and Tennant, 1988).

**TABLE 4:** The structural alerts extracted from the antiseizure medications.

Toxicological endpoints	SA	SMARTS	Chemical Structures of AEDs						
Genotoxiccarcinogenicitymutagenicity	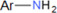	[a!r0][NX3H2]+D2:F5D16D2:F4D2:F6D2:F8	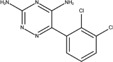 Lamotrigine	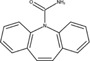 Carbamazepine	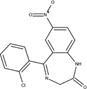 Clonazepam	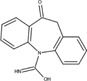 Oxcarbazepine	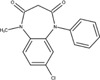 Clobazam	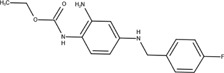 Ezogabine	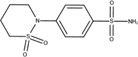 Sulthiame
	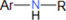								
	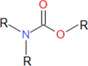	[NX3]([C,#1])([C,#1])[CX3](=[OX1,Sv2X1])[OX2,Sv2X2]C	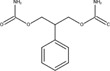 Felbamate	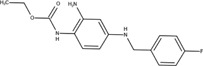 Ezogabine	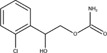 Carisbamate	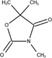 Trimethadione	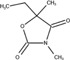 Paramethadione		
	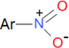	[NX3]([C,#1])([C,#1])[CX3](=[OX1,Sv2X1])[OX2,Sv2X2]C[a!r0][$([NX3+](=[OX1])([O-])),$([NX3](=O)=O)]	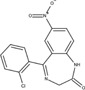 Clonazepam						
	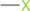	[CX4][Cl,Br,I]	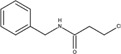 Beclamide						
Idiosyncratic toxicity	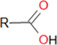	[#6][CX3](=[OX1])[OH1]	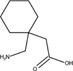 Gabapentin	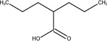 Valproate	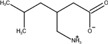 Pregabalin	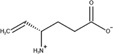 Vigabatrin	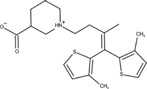 Tiagabine		
	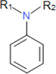	c1ccccc1[NX3]([#1,CX4!R,$(c1ccccc1),$([CX3](=[OX1])[#6]),$([CX3](=[OX1])[OX2][#6	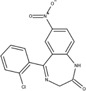 Clonazepam	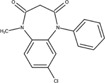 Clobazam	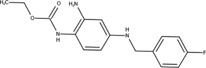 Ezogabine				
	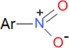	[a!r0][$([NX3+](=[OX1])([O-])),$([NX3](=O)=O)]	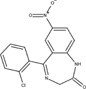 Clonazepam						
	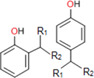	$(c1([OH1])c([CX4]([#1,#6])[#1,#6])cccc1),$(c1([OH1])ccc([CX4]([#1,#6])[#1,#6])cc1)]	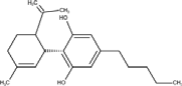 Cannabidiol						
	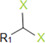	[$([CX4]([F,Cl,Br,I])([H,!F!Cl!Br!I])([H,!F!Cl!Br!I])[H,!F!Cl!Br!I]),$([CX4]([#6])([F,Cl,Br,I])([F,Cl,Br,I])[H,!F!Cl!Br!I])]	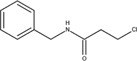 Beclamide						
	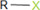	c1ccc[sX2]1	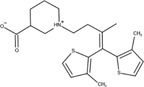 Tiagabine						
Potential electrophilic gents	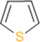	[#6][CH2][NX3H2]						
	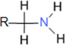	[$([cH]1cc(=[O,NH])cc[o,n]1),$([cH]1cc(=[O,NH])[o,n]cc1),$(c1cc(=[O,NH])[o,n][cH]c1)]	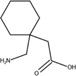 Gabapentin	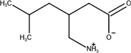 pregabalin					
	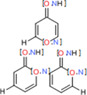	c1([CX3!R]=[CX3!R])ccccc1	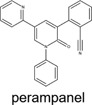 perampanel						
	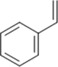	[CH3,$([CH2]([F,Cl,Br,I])[#6]),$([CH]([F,Br,Cl,I])([#6])[#6]);!$([CH2,CH]C=O)	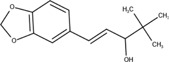 stiripentol						
	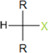	$([CH2,CH][CX4][NX3,SX2]);!$([CH2,CH][CX4][F,Cl,Br,I])][F,Cl,Br,I]	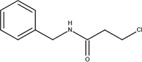 Beclamide						
Endocrine disruption	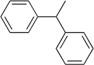	c1cc(ccc1)-[#6](-c2ccc​cc2)-[#6]	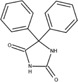 phenytoin	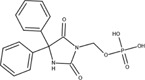 fosphenyoin					

At present, three third-generation antiepileptic drugs, namely perampanel, rufinamide, and brivaracetam were used in clinics and there is still insufficient data to support the risk assessment of teratogenicity. The predicted probability of teratogenicity was 0.80, 0.75, and 0.50 for perampanel, rufinamide and brivaracetam, respectively, indicating a high risk of perampanel and rufinamide and a moderate risk of brivaracetam. In the FDA drug labeling, the evidence provided by animal studies showed that these three ASMs had developmental toxicity in pregnant rats or rabbits at clinically relevant doses or at plasma exposures greater than clinical exposures, including visceral abnormalities, skeletal abnormalities, decreased fetal weight and embryo-fetal death for perampanel exposure at dose of 1, 3 or 10 mg/kg/day, skeletal abnormalities, decreased fetal weight and embryo-fetal death for rufinamide exposure at dose of 20, 100, and 300 mg/kg/day, and embryo-fetal death and decreased fetal weight only for highest dose brivaracetam exposure. One case report reported 2 cases of minor congenital malformations in the offspring exposed to brivaracetam during pregnancy (Paolini et al., 2020). As a result, our model can be used as a tool to alert new drugs that still lack clinical evidence for teratogenicity.

Some caveats of this study were worth further discussing. It is still a big challenge to develop a reproducible procedure to assess the teratogenic risk for the drugs. A set of attributes, such as severity, expectedness and causality, should be considered in annotating the teratogenic risk for a drug. In this study, we annotated the teratogenic risk for the drugs based on the FDA-approved drug labeling by considering the fact that drug labeling is a compilation of up-to-date drug safety information and a relatively accurate and stable data source. Consequently, we annotated 67 drugs with teratogenic risk (positive samples) and 45 drugs with low teratogenic risk (negative samples). The average precision achieved by our model in 1,000 iterations of 5-fold cross-validation was 0.690. When validating the model by using the FAERS data, we can only determine the drugs with teratogenic risk when the teratogenicity is reported. As a result, we only tested the recall rate of our model. Note that the annotation was based on the US FDA-approved drug labeling. The classification of the drugs might be different when using the drug labeling of other countries. The teratogenic risk of the drugs used in the modeling is determined by the FDA drug labeling. In the drug list, all of the 26 drugs in the therapeutic category of antineoplastic and immunomodulating agents (L) were assigned to the high teratogenic risk group (positive samples). It may be one of the main reasons that 92.3% of drugs were predicted to have teratogenic risk when using the FAERS dataset to validate the predictive model. In comparison, the number of drugs in the therapeutic category N (Nervous system) in the high teratogenic risk group (positive samples) and the low teratogenic risk group (negative samples) is relatively balanced (7 positive and 10 negative samples). As a result, 53.8% of the drugs in the FAERS dataset were predicted to be teratogenic. When more balanced data can be obtained with the update of the FDA drug labeling, the predictive performance of our model can be further improved. Our proposed model only based on the chemical structures of the drugs. Although it is simple and easy to use, the limited information restricted the predictive performance of the model. Considering that drug-induced malformations are usually dose-dependent and affected by many factors. Additional information, such as the cell-based *in vitro* data, the data from animal studies as well as the pharmacogenomics data, involved in the modeling procedure may improve the model performance. Furthermore, a comprehensive model is also needed.

## Conclusion

In this study, we proposed a SAR model for predicting the potential teratogenic risk of the ASMs based on the chemical structures of the drugs. The model was sensitive to detecting the high teratogenic risk drugs. Our findings can be helpful for the prevention and early detection of drug-induced teratogenesis.

## Data Availability

The datasets presented in this study can be found in online repositories. The names of the repository/repositories and accession number(s) can be found in the article/Supplementary Material.

## Abbreviations

ASMs, antiseizure medications; FAERS, Spontaneous Adverse Events Reporting System; LR, logistic regression; MCMs, major congenital malformations; ROR, reporting odds ratio; SAR, structure-activity relationship; SAs, structural alerts; SVM, support vector machine; RF, random forest; XGBoost, extreme gradient boosting.
