# Supplementary figures and images for: Structure-Activity Relationship (SAR) Model for Predicting Teratogenic Risk of Antiseizure Medications in Pregnancy by Using Support Vector Machine
Source: Front Pharmacol. 2022 Feb 25;13:747935. doi: 10.3389/fphar.2022.747935 (PMC8914116; doi:10.3389/fphar.2022.747935)

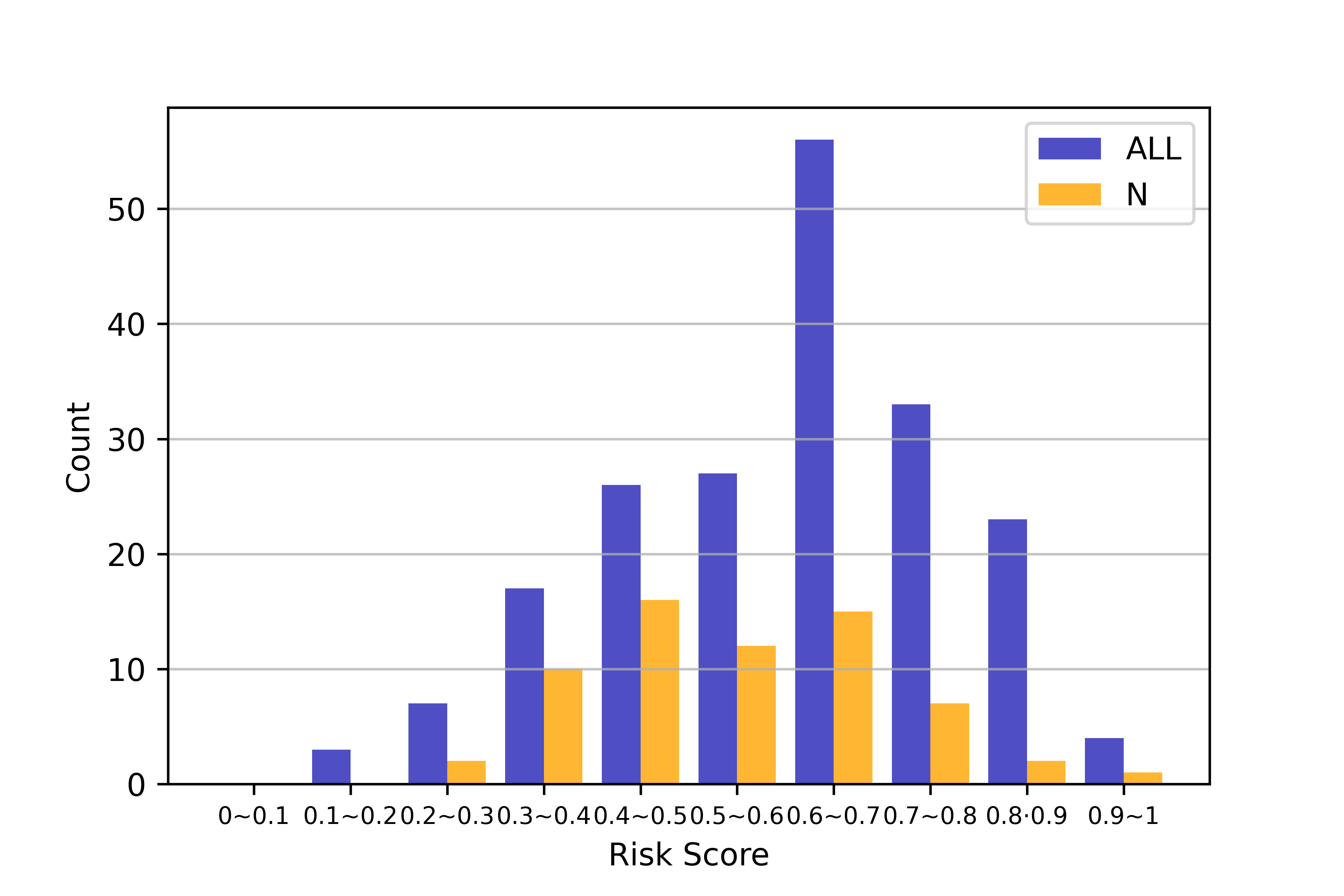

Supplement: Supplementary file 2 [file Image1.JPEG]
