# Supplementary material for: Structure-Activity Relationship (SAR) Model for Predicting Teratogenic Risk of Antiseizure Medications in Pregnancy by Using Support Vector Machine
Source: Front Pharmacol. 2022 Feb 25;13:747935. doi: 10.3389/fphar.2022.747935 (PMC8914116; doi:10.3389/fphar.2022.747935)
Supplement: Supplementary file 4 [file DataSheet1.docx]

**Supplementary Figure 1. The distribution of the predicted probability of the teratogenic risk of the drugs in FAERS dataset.** ALL represents all drugs and N represents the Nervous system drugs among them.

Supplementary Table 1. The numbers of cases of each specific single congenital malformation and the number of cases of multiple congenital malformations induced by 22 antiseizure medications (ASMs). The data are summarized from FAERS database.

Supplementary Table 2. The preferred terms considered for case retrieval in FAERS.
